# Biophysical models resolution affects coral connectivity estimates

**DOI:** 10.1038/s41598-023-36158-5

**Published:** 2023-06-09

**Authors:** Antoine Saint-Amand, Jonathan Lambrechts, Emmanuel Hanert

**Affiliations:** 1 Earth and Life Institute (ELI), Université catholique de Louvain, Croix du Sud 2, 1348 Louvain-la-Neuve, Belgium; 2 Institute of Mechanics, Materials and Civil Engineering (IMMC), Université catholique de Louvain, Avenue Georges Lemaître 4-6 1348 Louvain-la-Neuve, Belgium

**Keywords:** Physical oceanography, Ecological modelling, Ecological networks, Coral reefs

## Abstract

Estimating connectivity between coral reefs is essential to inform reef conservation and restoration. Given the vastness of coral reef ecosystems, connectivity can only be simulated with biophysical models whose spatial resolution is often coarser than the reef scale. Here, we assess the impact of biophysical models resolution on connectivity estimates by comparing the outputs of five different setups of the same model with resolutions ranging from 250 m to 4 km. We show that increasing the model resolution around reefs yields more complex and less directional dispersal patterns. With a fine-resolution model, connectivity graphs have more connections but of weaker strength. The resulting community structure therefore shows larger clusters of well-connected reefs. Virtual larvae also tend to stay longer close to their source reef with a fine-resolution model, leading to an increased local retention and self-recruitment for species with a short pre-competency period. Overall, only about half of the reefs with the largest connectivity indicator values are similar for the finest and coarsest resolution models. Our results suggest that reef management recommendations should only be made at scales coarser than the model resolution. Reef-scale recommendations can hence only be made with models not exceeding about 500 m resolution.

## Introduction

Coral reefs have the highest biodiversity among marine ecosystems and are the foundation of complex food chains^1,2^. While covering only 0.5% of the seafloor, they are home to about a third of all marine species. Corals provide habitat, shelter, nursery areas and food to many marine animals and plants. They also provide essential ecosystem services such as the protection of coastlines against storms as well as food and income to local communities^2^. However, corals dramatically declined over the past few decades^3–5^. Their decline has been driven by several anthropogenic stressors, at the global and local scales^6–10^. Among those stressors, global warming led to a sharp increase in coral bleaching events, which are now occurring almost on a yearly basis in some parts of the world^11–13^. During bleaching events, endosymbiotic algae leave their coral host, which then looses its principal source of energy. If the bleaching event lasts too long, corals eventually die, with an increased prevalence of post-bleaching mortality observed in recent years^10,14–16^.

Following disturbances, the exchange of coral larvae between reefs plays a crucial role in repopulating damaged reefs^17–19^. Because adult corals are physically attached to their home reef, the pelagic larval phase is the only moment when an exchange can occur between any two reefs. Those exchanges are happening during yearly mass spawning events and involve the dispersal of a very large number of larvae. Understanding how and where those larvae disperse is essential to apprehend the dynamics and resilience of coral reefs^20^. However, current Marine Protected Areas (MPA) only partially take connectivity estimates into account^17,18,21,22^. The integration of connectivity estimates in management planning is however not trivial. Larval dispersal is indeed very difficult to directly observe or measure because of the small size of larvae, the number of species at stake and the vastness of coral ecosystems^23^.

With the increase in computational resources, biophysical models have become a popular tool to study connectivity. Those models are able to simulate the transport and evolution of larvae driven by the ocean currents and their specific biological traits. Biophysical models can be used to study coral connectivity over spatial scales larger than the size of entire coral reef ecosystems, hence making them good candidates to inform reef management. The ability of models to correctly represent connectivity, and more specifically identify resilient reefs, has however been recently debated^24–26^. In this controversy, one issue concerned the spatial resolution required to effectively capture all the major processes happening around the reefs. If the resolution is coarser than the size of a reef, it will not correctly reproduce the reef-scale hydrodynamic features that influence larval dispersal just after spawning. This question appears to be central to assess the ability of biophysical models to represent connectivity patterns. Vasile *et al.*^27^ argued that the examination of the quality of hydrodynamic models is often neglected, especially in larval dispersal studies, even if those models are deemed to be critical to study dispersal patterns.

Only a few studies have investigated the impact of model resolution on larval dispersal and none of them have considered model resolutions fine enough to be applicable to coral reefs. Mitarai *et al.*^28^ previously showed that larval dispersal in the coastal ocean is influenced by local circulation patterns for time periods of 30 days or less, but they limited their analysis on coastal release sites. Putman and He^29^ observed a greater offshore transport for sea turtles dispersal with coarser spatio-temporal resolution, but the finest level of detail they achieved was at a scale of about 6–9 km with daily outputs. Several studies showed that using high resolution models improves the simulation of sub-mesoscale flows^30–34^, but most of them only noted local differences with conversely no strong impact on the global dispersal patterns. Dauhajre *et al.*^35^ performed a detailed sensitivity analysis of the influence of the spatial resolution on hydrodynamic models outputs. They considered resolution from 36 m up to 1 km, and observed a stronger retention for lower resolution models. Their analysis was however limited to only 19 nearshore sites. They argue that resolving small-scale currents on the shelf is potentially a necessity for accurate simulations of particle transport in the nearshore. Conversely, Bracco *et al.*^36^ observed an increased diffusivity for coarser 9 km resolution models compared to their fine 1 km resolution ones. Colberg *et al.*^37^ compared two 3D ocean models of the Great Barrier Reef (Australia), with a horizontal resolution of 500 m and 4 km respectively. They found that the 1 km resolution model better represented the sub-surface temperature and salinity in the deep ocean. They also observed better current directions because of the improved bathymetry representation on the 1 km grid, but did not notice major differences in current speed. However, they did not investigate how such discrepancies could impact larval dispersal.

Here we evaluate the impact of a biophysical model spatial resolution on reef connectivity estimates. We consider an ocean model of the entire Great Barrier Reef (GBR), which is the largest coral reef ecosystem in the world, and run larval dispersal simulation between the thousands of reefs composing the GBR. To simulate the ocean circulation, we use the multiscale coastal ocean model SLIM (”Second-generation Louvain-la-Neuve Ice-Ocean Model”, https://www.slim-ocean.be), which relies on unstructured meshes to smoothly adapt the spatial resolution to the coastal topography. SLIM was first applied to the GBR by Lambrechts *et al.*^38^, and then used for several connectivity and dispersal studies^20,39–46^. In this study, we simulated coral larval dispersal driven by the currents computed on five different meshes whose finest spatial resolution ranges from 250 m to 4 km. We then analyzed the resulting connectivity estimates with graph theory indicators and community detection algorithms to quantify the influence of the underlying model resolution.

## Results

To investigate the impact of model resolution on larval dispersal and connectivity, we released $\sim$ 12 millions particles over $\sim$ 3000 reefs for two coral species (*Acropora millepora* and *Goniastrea retiformis*) and simulated their trajectories with five different model resolutions. On most of the figures presented in this section, the five model setups are identified with their highest resolution. For instance, “250 m”, refers to connectivity results obtain with a biophysical model at the finest 250 m resolution, whereas “4 km” refers to the results derived at the coarsest 4 km resolution.

### Effect of model resolution on larval release

**Figure 1:**
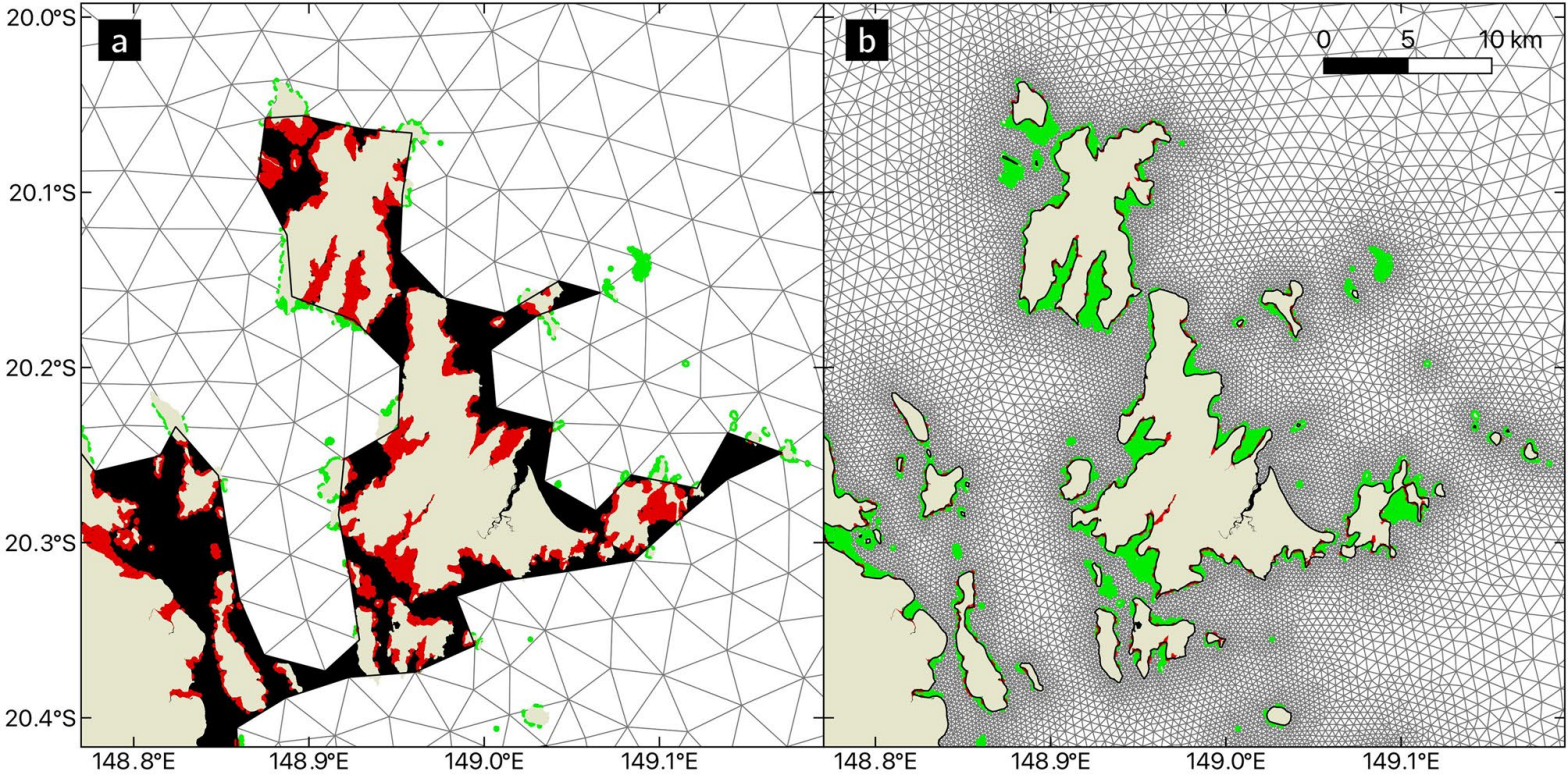
Depending on the model resolution, the coastlines accuracy varies. This leads some reefs to fall partially or entirely out of the domain. This figure covers the Whitsundays, identified with a red square on Fig. 7. Red zones depict (parts of) reefs falling out of the domain on (**a**) the coarsest and (**b**) finest mesh, whereas green areas correspond to reef parts inside the domain. The original land map is represented in beige and mesh triangle elements in white. The black zones display sea areas that are not covered by the mesh.

Discrepancies between models with different resolutions already appear at the start of the simulation, when larvae are released. Using a coarse resolution model leads to a poor representation of the coastline topography. Hence, some reefs fall partially or totally out of the modeling domain (Fig. 1): more than 5% of the reefs are not represented at all on the coarsest mesh, but this fraction linearly drops to less than 0.2% on the 250 m mesh. Consequently, the number of particles released inside the domain increases as the resolution becomes finer: the fraction of released larvae increases linearly from 98.7 to 99.8% between the coarsest and the finest resolutions. When generating the meshes, we prevented them from overlapping land. Another strategy would have been to constrain the meshes to include all coastal reefs at the expense of overlapping land and hence removing islands smaller than the mesh resolution from the computational domain.

### Effect of model resolution on larval dispersal

**Figure 2:**
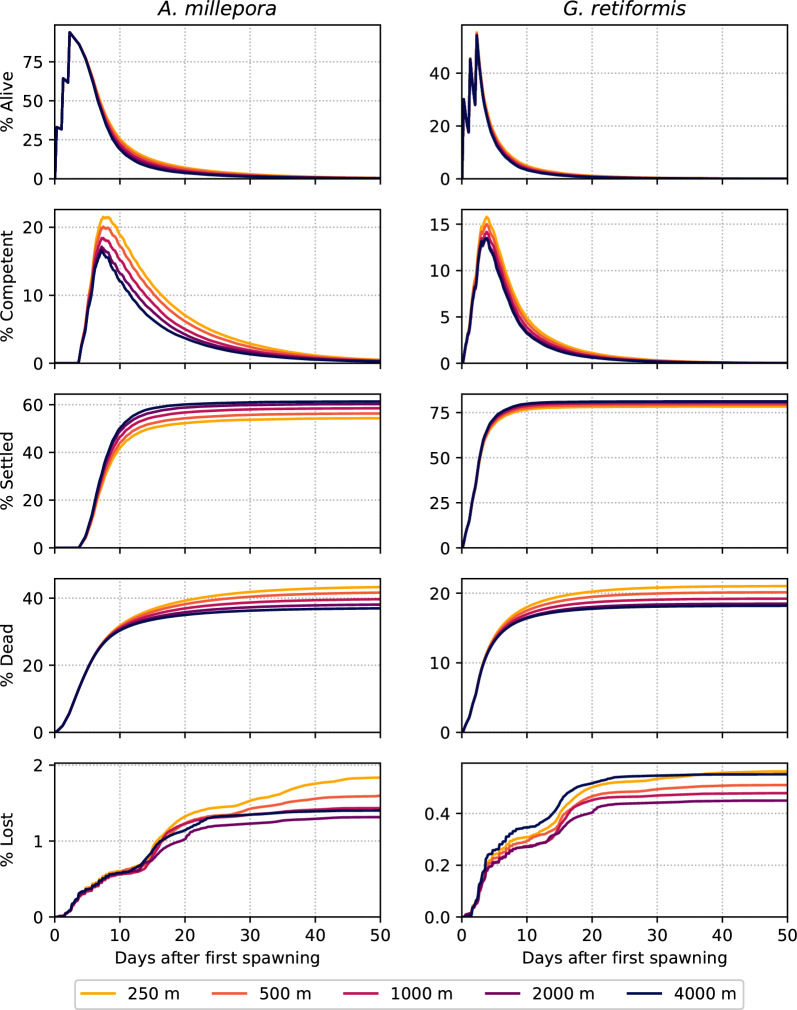
Percentage of larvae in each possible state, namely alive in the water column, alive in the water column and competent, settled, dead, or lost outside the modeling domain. For both species, larvae tend to settle faster on average for coarser resolution models, leading to more dead particles for finer resolution models. The differences between resolutions are however larger for *A. millepora* than for *G. retiformis* due to the absence of precompetency period for this second species.

The evolutions of the larval states during the simulation is quite similar for the different resolutions (Fig. 2). Firstly, the number of larvae alive in the water column follows about the same trend on every mesh for each species. The three jumps at the beginning of the simulation correspond to the three spawning events. At this stage, the influence of larval biological parameters appears in the fraction of larvae alive in the water column over time: as *G. retiformis* has no precompetency period, some larvae can settle as soon as they have been released. The sharp drops in the fraction of drifting larvae between spawning events are hence explained by the settlement of larvae.

There are however also some differences in the larval dynamics between simulated with different resolutions: on average, larvae tend to settle faster on coarser resolutions (Fig. 2). This can be related to currents representation on the different meshes: with slower water flow over reefs and more intense eddy activity on finer meshes, larvae will take a little longer to leave their source reef, delaying the time to reach another reef to settle. On finer meshes, the reefs’ geometry is more finely represented, hence yielding a better representation of the spatial variations of the bottom drag. As a result, the flow will be deflected by the reefs. This, in turn, impacts the larvae dispersal pathways as larvae will move more slowly over the reefs and more quickly around them (Fig. 7c,d).

### Effect of model resolution on connectivity indicators

**Figure 3:**
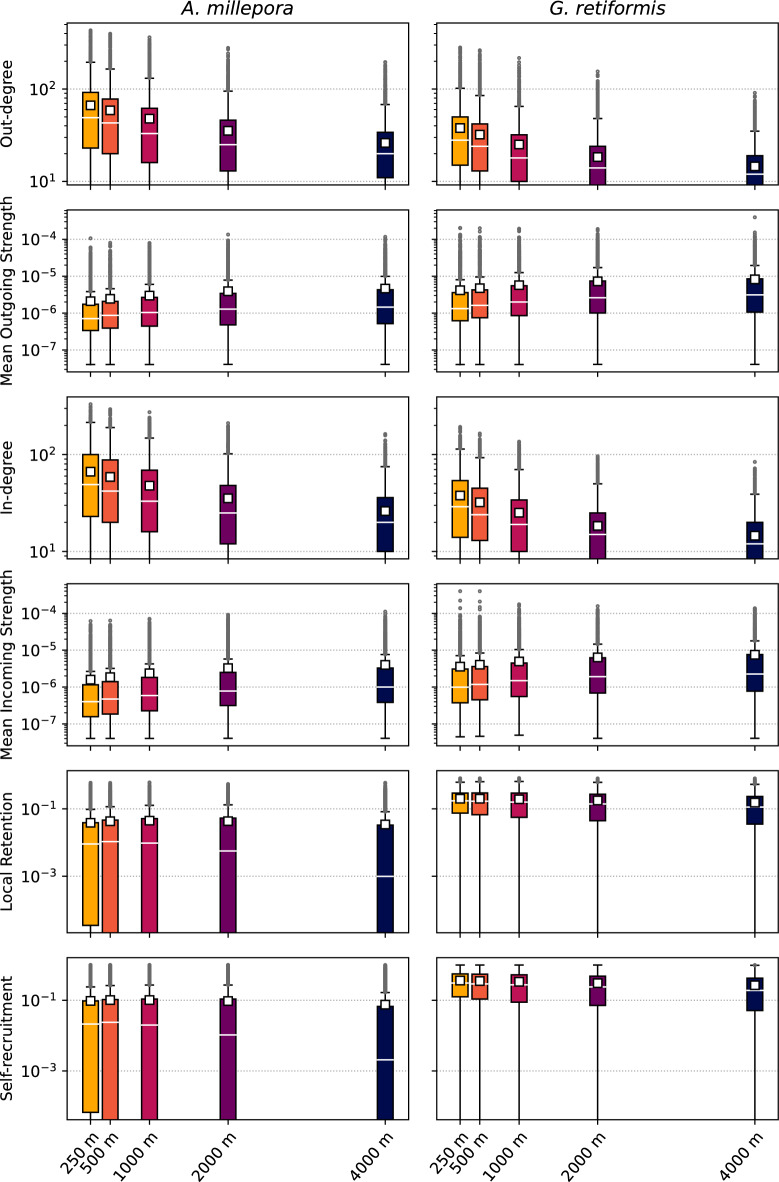
Distribution of the six connectivity indicators for the five simulations, identified by their highest resolution. The white squares represent the mean value. A trend is observed for each indicator, except for the local retention and self-recruitment of *A. millepora*. This linear trend shows that the model resolution has an impact on the simulated connectivity.

**Figure 4:**
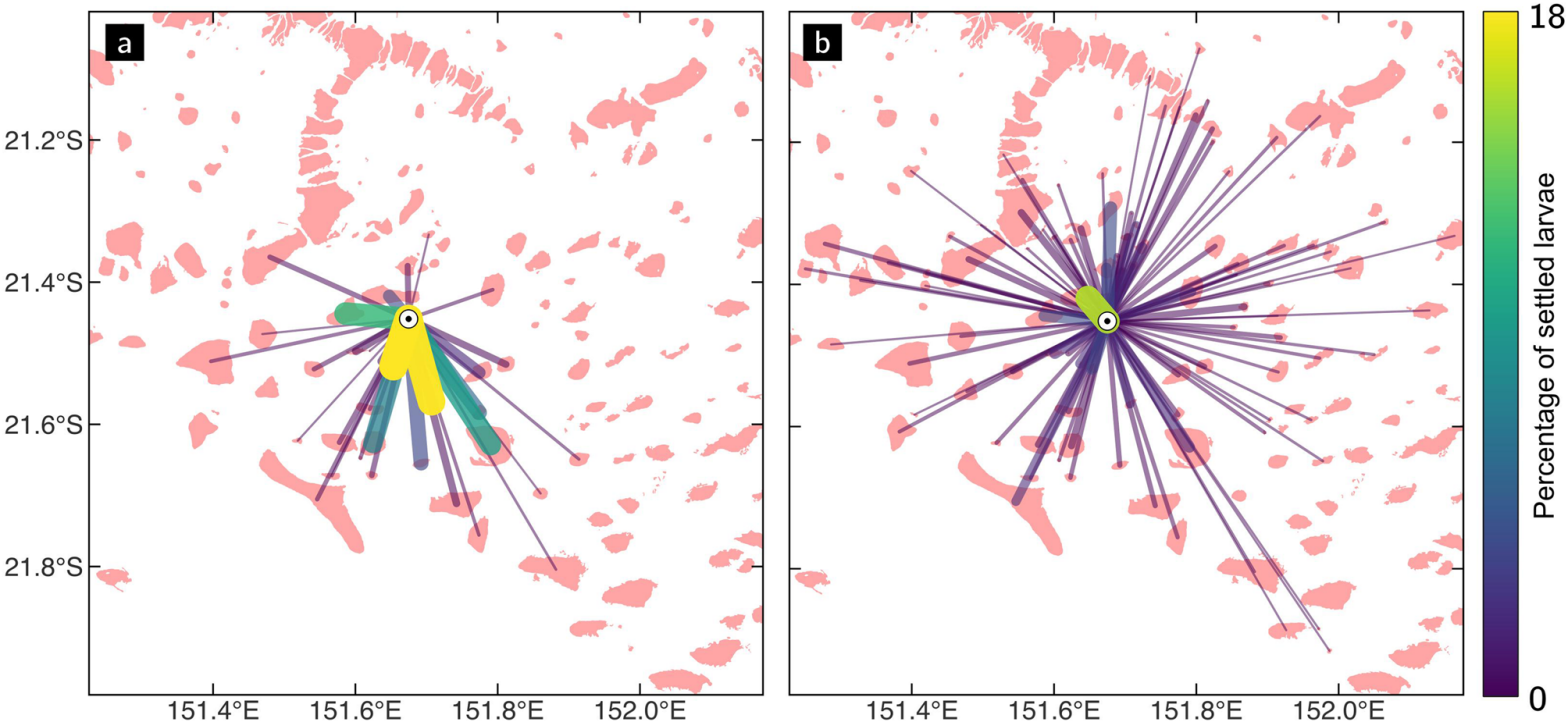
Outgoing connections from one particular reef for *G. retiformis* larvae colour-coded by their strength (i.e., the number of virtual larvae exchanged, normalized by the total number of settled larvae), as simulated on (**a**) the coarsest 4 km and (**b**) the finest 250 m resolution. Reefs are represented in pink. The total number of larvae exchanged is about the same in both cases. However, there are more connections of weaker strength on the finest resolution.

Incoming and outgoing connectivity indicators are influenced by the underlying model resolution. As it becomes finer, we observe an increasing number of connections and a decreasing mean connection strength, both for the incoming and outgoing connectivity (Figs. 3 and 4). This trend is observed for both species, even if *G. retiformis* displays less connections of greater strength compared to *A. millepora*. The shorter dispersal time of *G. retiformis* reduces the average distance separating the source and destination reefs (less than 10 km for *G. retiformis* compared to more than 20 km for *A. millepora*). The radius of influence is hence reduced for *G. retiformis*, explaining its lower in- and out-degree values.

Both species clearly differ for local retention and self-recruitement (Fig. 3). For *A. millepora*, both indicators show similar distributions across all resolutions. In contrast, they both decrease for *G. retiformis* as the resolution becomes coarser. For this second species, reefs appear, at the same time, more self-resilient and more isolated when using a high resolution model. The absence of a precompetency period for *G. retiformis* explains this difference between species: a larger fraction of the larvae released on a reef will settle on the same reef. The major part of the settlement is happening during the first hours and days of simulation following spawning. Half of the *G. retiformis* larvae settle in less than three days since the first spawning (Fig. 2). The dispersal time for those larvae is therefore very short, preventing them from traveling away from their source and increasing the probability of settling where they were produced. Larger values on fine meshes can be explained by the more turbulent hydrodynamics in the direct vicinity of reefs that tends to retain larvae close to their source reef.

Distributions of connectivity indicators have been statistically compared. As those distributions are not normally-distributed, we used the non-parametric Friedman test to run the comparisons. For each indicator of both species, the results of this test were highly significant ($p<0.001$), denoting that the indicator distributions are not similar among all resolutions.The effect size, or the agreement between values of indicators on each mesh, was computed with the Kendall’s coefficient of concordance (W). This coefficient showed contrasting results: while we observed a large concordance for the in- and out-degree as well as for the mean incoming strength (0.45 < W < 0.6), the agreement was only moderate for the mean outgoing strength (0.2 < W < 0.3), and even low for local retention and self-recruitement (W < 0.1).

The analysis was further refined by detecting potential trends thanks to one-sided Wilcoxon signed-rank tests. Each pair of indicator distributions computed with models of successive resolutions was individually compared, and a Bonferroni correction was applied on the obtained p-values to counteract the multiple comparisons’ problem. For *A. millepora* local retention and self-recruitement indicators, the comparison of distributions for successive resolutions gave significant results ($p<0.05$), but no trend appeared as the tests consecutively indicated smaller and larger distributions. For all other indicators, comparisons between consecutive resolutions were either non-significant (only for the comparison of *G. retiformis* local retention between the 250 and 500 m resolutions), or significant in the same direction systematically (in any other case). Hence, except for *A. millepora* local retention and self-recruitement, Wilcoxon tests highlight a linear trend between the model resolution and the distributions of connectivity indicators.

**Figure 5:**
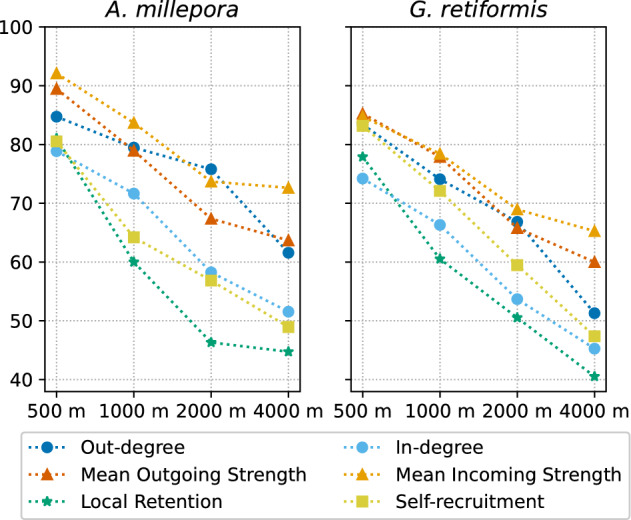
Percentage of reefs whose connectivity indicators’ values are among the top 5% values on both the finest 250 m resolution and the other resolutions. Only about half of the connectivity hotspots derived from the finest 250 m and coarsest 4 km models are the same.

Discrepancies between model resolutions further increase when considering the top 5% hotspots for each connectivity indicators (Fig. 5). These hotspots are often considered in studies making reef management recommendations. Between the 250 m and the 500 m resolutions, 78–92% (*A. millepora*) and 74–84% (*G. retiformis*) of the connectivity hotspots are the same. Those ranges respectively decrease to 45–72% and 41–66% on the coarsest resolutions for both species. Discrepancies between coarse and fine resolutions are the largest for local retention and self-recruitment (more than 50% discrepancy). This highlights again the impact of the reef-scale hydrodynamics on the larvae dispersal after the release. Discrepancies of hotspots are also consistently slightly lower for *G. retiformis* than for *A. millepora*. The distribution of the in- and out-degree and strength hotspots along the GBR reveals some regional clustering (see Figs. S.1 and S.2 in Supplementary Material). The in- and out-degree hotspots are nearly all located offshore in the southern GBR while the mean strength hotspots are mostly found in the northern and central part of the GBR. Local retention and self-recruitement hotspots are however more scattered along the entire GBR. These regional similarities hence do not translate into similarities at the local scale.

### Effect of model resolution on community detection

**Figure 6:**
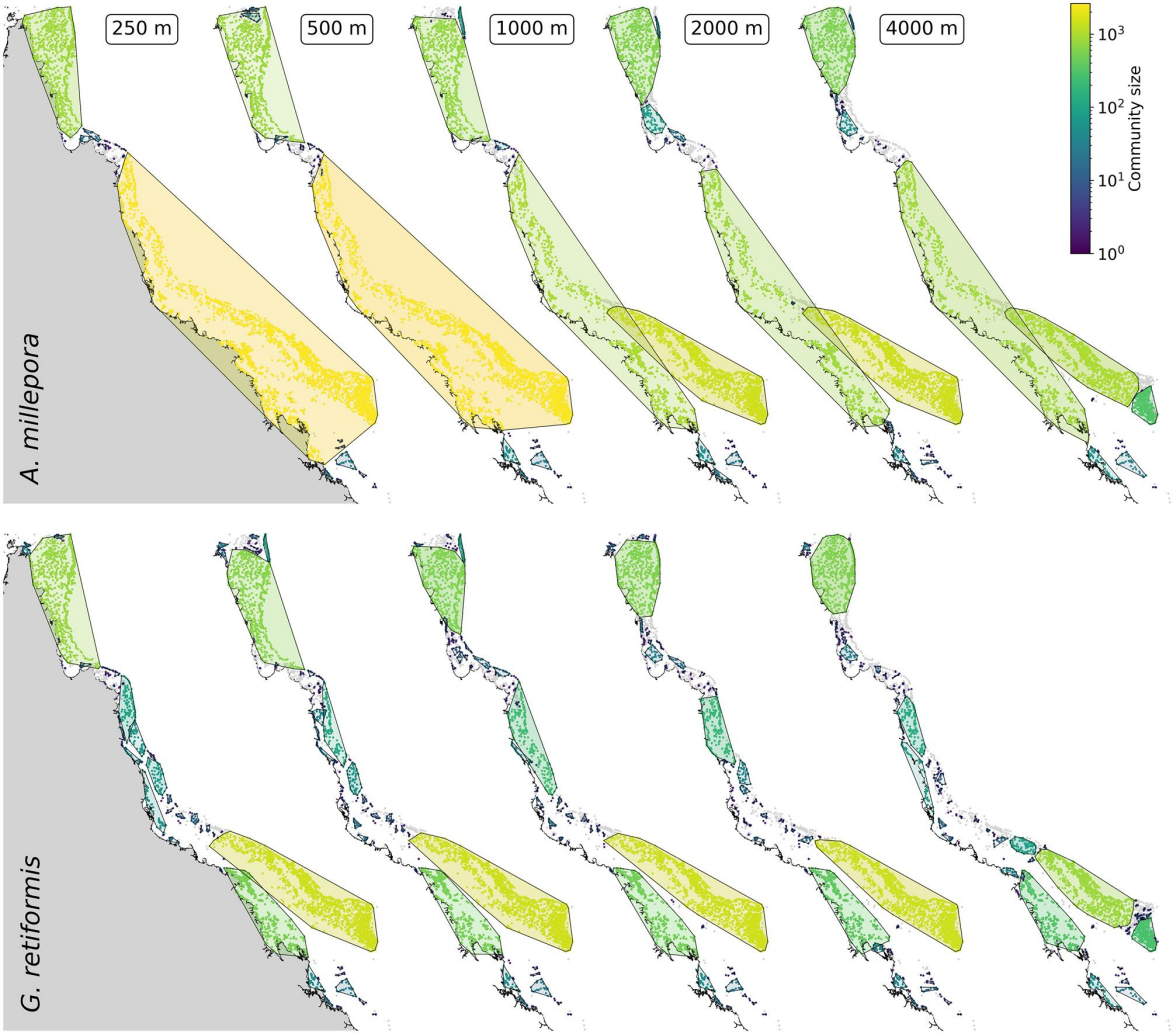
Communities of strongly connected reefs as detected for the five model resolutions. Each community is colour-coded by the size (i.e., the number of reefs included). Convex-hulls encompassing all reefs belonging to the same community are also drawn. As the spatial resolution decreases, the community structure appears more fragmented, with more smaller communities.

The southern, central and northern parts of the GBR explicitly appear in the community structure (Fig. 6). There is always a community in the northern GBR. The transition between the northern and the central parts seems to stand as a frontier to bidirectional connectivity. This is probably due to the large-scale currents from the Coral Sea entering the GBR at those latitudes and causing strong diverging flows to the North and the South in this region. In the centre, coral reefs are further apart, which results in a patchwork of relatively small communities for the fast-settling *G. retiformis* species. Conversely, for *A. millepora*, reefs in the central GBR are well-connected with the southern reefs, forming a large community that includes both offshore and nearshore for the 250 m and 500 m resolution models and only nearshore reefs for coarser models.

If the three main parts of the GBR appear in the community structure for each model resolution, this community structure becomes fragmented when the resolution coarsens (Fig. 6). This is observed for both species. The number of communities containing at least three reefs hence jumps from 15 to 25 for *A. millepora* between the finest and the coarsest resolutions, and from 44 to 76 for *G. retiformis*. As the resolution coarsens, we observe a similar trend for the number of reefs not included in any community: from 162 to 819 single reefs for *A. millepora*, and from 231 to 839 for *G. retiformis*. On finer resolutions, in- and out-degree values are higher (Fig. 3), meaning that the connectivity graph is denser, i.e. there are more connections between pairs of reefs. This results in more reefs belonging to the same community.

When comparing both species, we observe more smaller communities for *G. retiformis* than for *A. millepora*. This is in agreement with the smaller in- and out-degree values obtained for *G. retiformis* (Fig. 3). The shorter dispersal time indeed reduces the probability of having closed connectivity pathways for *G. retiformis*, which are necessary to group reefs in the same community. However, the community fragmentation trend as the resolution coarsens remains valid for both species. For *A. millepora*, on 250–500 m resolutions, most of the southern offshore and nearshore reefs belong to the same community. With coarser resolutions, offshore and nearshore reefs are however separated in two distinct communities. For *G. retiformis*, as there is no precompetency, the distinction between offshore and nearshore reefs appears for each model resolution. Similarly, for both species, the offshore southern reefs are always grouped together in one big community except on the coarsest resolution, where the southern tip of the offshore reef system is disconnected form the main community.

## Discussion and perspectives

In this study, we have shown that, all other things being equal, the spatial resolution of a biophysical model influences the resulting dispersal patterns and connectivity metrics. We therefore suggest that the scale at which a model can provide recommendations for reef management, for example regarding the delineation of Marine Protected Areas^47,48^ or the identification of the most suitable reefs for restoration measures^49,50^, can not be finer than its spatial resolution. Any recommendation based on model results is therefore valid and applicable at scales coarser than that resolution. For instance, models with a 4 km resolution should not be used to make recommendations at scales finer than about 10 km. However, at this scale, all the complexity of a reef system is lost and the identification of the reefs with the best connectivity properties is compromised. This is however what many connectivity studies are trying to do as, for instance, the best source reefs can be targeted for protection or restoration actions as they contribute more to the overall resilience of the entire coral reef ecosystem. Yet, the high sensitivity of the connectivity hotspots to the model resolution suggest to be very cautious when interpreting results derived with a coarse resolution and using them to make management recommendations. Bode *et al.*^25^ made similar suggestions and urged to provide clear caveats with the management recommendations inferred from connectivity simulation.

Reef-scale management programs should hence not rely on coarse resolution model outputs. Yet, some recent coral connectivity studies were based on models with a 4 km resolution, hence too coarse to resolve the reef-scale dynamics^24,51^. Instead of considering the individual reef extents, those connectivity studies rely on coarse grid pixels that are tagged as ”containing” or ”not containing” reefs. As a consequence, connectivity results cannot be interpreted at the scale of individual reefs, but only at the scale of ”patch of reefs”, which depends on the resolution of the underlying biophysical model. Such a workaround is however source of great simplification because it ignores the complexity of dispersal patterns at the reef scale, which might bias connectivity estimates and make them not suitable for management purposes. We therefore strongly recommend to carefully select and parametrize models to ensure they are able to capture the scales at which the processes of interest take place. In particular, for reef-scale management recommendations, any model running at a spatial resolution coarser than about 500 m should be dismissed. Models are indeed only valid for a specific range of scales that depends both on their mathematical formulation and on their resolution. Any conclusions drawn at scales out of that range—and specifically at scales smaller than the model resolution—should be questioned.

The key focus of this work was precisely to demonstrate that the hydrodynamic model resolution impacts coral connectivity estimates both at the reef and regional scales. All our connectivity results suggest that ocean circulation patterns are more complex and lead to less directional dispersal patterns when simulated with a fine 250–500 m resolution model. At that resolution, the more accurate representation of the bathymetry and reef topography yields a more detailed representation of the dispersal processes. Conversely, at coarser resolutions, currents are more uniform and can hardly capture the reef-scale dynamics. Connectivity estimates derived from biophysical model simulations are hence sensitive to the model resolution. The in- and out-degree indicators show that there are more in/out connections per reef on the finest resolution models, but these connections are weaker (Fig. 3). On the contrary, with the coarsest resolution models, there are fewer connections, but they carry more larvae. Because the transport is more dispersive, the communities of strongly connected reefs are larger with the finest resolution (Fig. 6). More connections indeed mean more possibilities for multistep connectivity pathways between reefs, and also more multidirectional connections.

**Figure 7:**
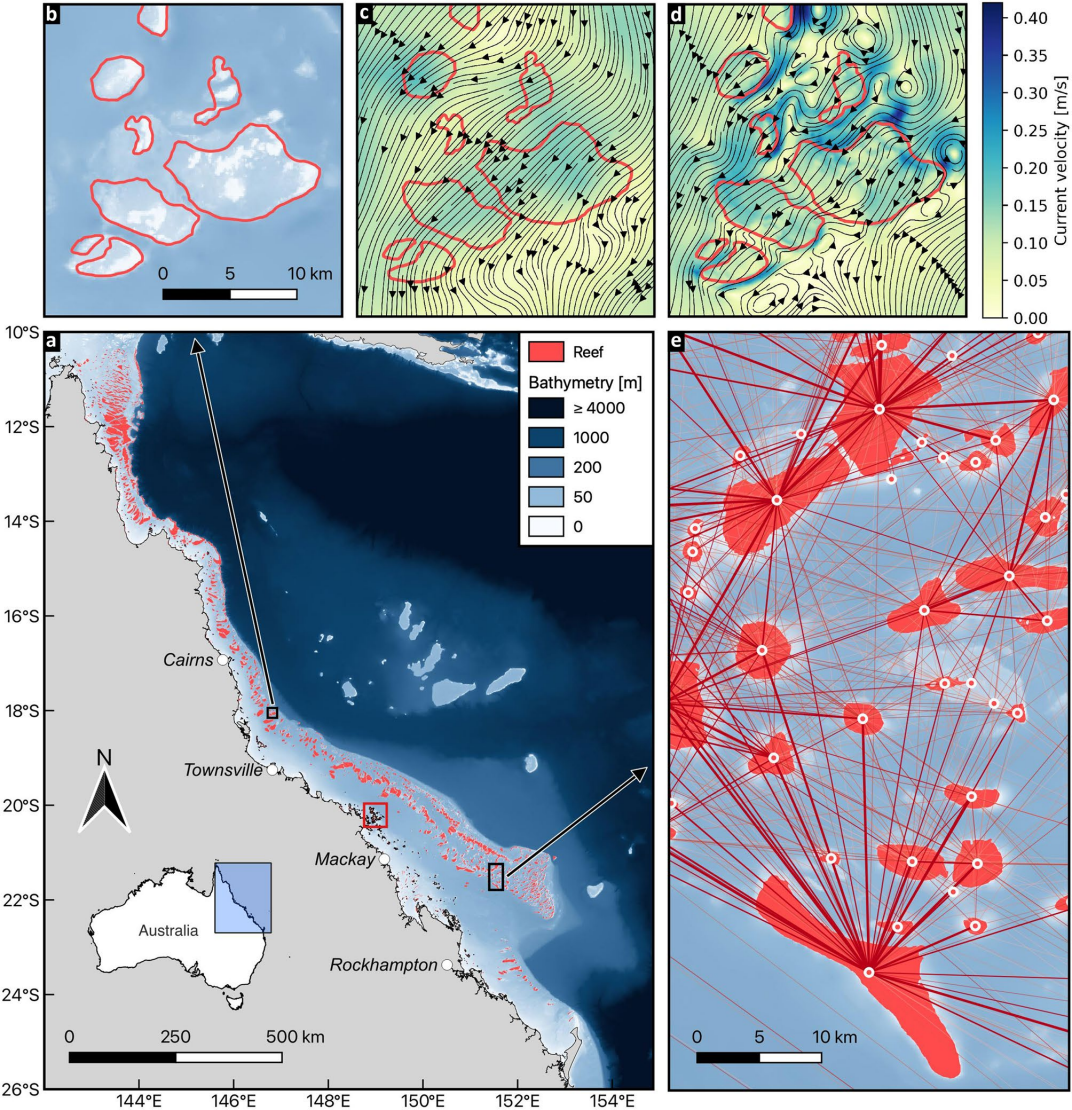
Overview of the study area. (**a**) The GBR is located on the continental shelf along the North-eastern coast of Australia. It is composed of more than 3000 reefs and displays an average depth of a few dozen meters. (**b**) Close-up view on some reefs in the central part of the GBR. The limits of reefs, as indicated in the GBRMPA reef map, are shown in red. (**c**, **d**) Streamlines and velocity fields of the simulated currents on 15 Dec. 2020 at 5pm with the coarsest (**c**) and finest resolution model (**d**). (**e**) Overview of the connectivity network of larval exchanges between reefs (the darker the line, the more larvae are exchanged). Connections are displayed between reef centroids, represented with white circles.

Our results suggest that reef-scale differences in the hydrodynamics are causing regional-scale differences in the dispersal patterns. The large-scale and long-term dispersal and connectivity of virtual larvae are indeed directly influenced by their dynamics following their release. This is in turn depends on the reef-scale hydrodynamic processes such as flow acceleration, deflection and recirculation, which can only be reproduced if the model resolution is finer than the scales at which those processes take place (Fig. 7). While differences between fine and coarse resolution hydrodynamic models are limited in the open ocean, they are significant around reefs and along the coastline^52^. The hydrodynamic model resolution therefore strongly influences the dispersal patterns and subsequent connectivity of virtual larvae.

As the model resolution particularly affects larval dispersal simulations over and around reefs, consequences are observed for both the early and late stages of the larval dispersal. At spawning, the model resolution already influences the release representation: the number of larvae effectively released is reduced for reefs located along the coast for coarser resolution models as some of them will fall partially or totally out of the model domain (Fig. 1). Besides, the early fate of particles successfully released is also influenced by the model resolution. Models with a resolution finer than the reef scale better represent the bottom rugosity variations between the reef and the sandy seabed. The simulated flow is then weaker over the reefs and more intense between them. The water flow dissipation over reefs is known to increase the water residence time, as previously described as the ”sticky water effect” by Wolanski and Spagnol^53^. This effect has been shown to efficiently trap larvae close to their source reefs in dense reef systems^54^. Coarse resolution models tend to smooth the flow variability by ”spreading” the reef rugosity beyond the reefs boundaries, hence in effect reducing it over the reefs and increasing it around them. This results in less shear and therefore less eddy activity. Consequently, a coarse resolution model will tend to quickly flush larvae away from the source reef and transport them with minimal deflections from surrounding reefs. With a finer resolution model, larvae take more time to be flushed away. Once they have left their source reef, they are carried away by more complex current patterns that include local flow acceleration between reefs and recirculation eddies in their wake, which presence has previously been shown to effectively retain larvae near their natal reef^55^.

Once larvae are competent and able to settle, the model resolution also influences their chances of settling. If the reefs topography and bathymetry are better represented with a fine resolution model, larvae will have fewer chances to travel over other reefs as they are transported away from their source reef. This means that they will also have fewer chances to eventually settle on another reef. With a fine resolution, the local circulation over and between the reefs is indeed better represented leading to weaker currents over shallow reefs and more intense currents between them. This better representation of the local flow conditions could transport most of the particles in the channels between reefs, preventing them from being transported over the reef and settling. Conversely, the flow is less deflected by the reefs when using a coarse resolution model. Such models yield more directional circulation patterns that can quickly transport larvae further away from their source reef, hence increasing the chances of reaching a reef where they can settle.

Likewise, the model resolution has a larger impact for coral species with a short or vanishing precompetency period such as *G. retiformis*. For those species, the hydrodynamics at the reef scale will play a larger role as larvae can potentially settle as soon as they have been spawned. Species with a longer precompetency period such as *A. millepora* can be transported away from the reef system, to the open ocean, before they become competent and are hence less influenced by the reef topography. Here we observe that local retention and self-recruitment indicators for *A. millepora* are similar for all model resolutions, while for *G. retiformis* both indicators display larger values for the finest resolutions (Fig. 3). Our results agree with Huret *et al.*^30^, who also observed an increased within-site retention with their finest resolution model. On the other hand, our results do not agree with Dauhajre *et al.*^35^, who showed that coarser-resolution models overestimate self-connection resulting in weaker and more retentive connectivity patterns. This study was however limited to nearshore connectivity between only 19 sites, while we consider here 3000+ reefs located both neashore and offshore.

Although we can be confident that fine resolution models generally produces more accurate hydrodynamics^52^, we cannot formaly prove that this translates to greater accuracy in connectivity simulations. This probably constitutes the main limitation of this study. Validating demographic connectivity results remains very challenging. Given the vastness of coral reef ecosystems like the GBR, the number of reefs composing them, and the huge number of larvae released during spawning events, it is impossible to directly validate our models by tracking individual larvae in the water. Recently, several studies have attempted to compare the outputs of larval dispersal simulations with genetic data^56–58^, with varying degrees of success. Yet, to the best of our knowledge, no study has been able to validate biophysical models with genetic data on a scale as extensive as that of the GBR so far. The simulated hydrodynamics, which drive larval dispersal, has however been properly validated^38,20,52^.

We also did not evaluate what the impact of additional mesh refinements on the connectivity metrics. Here we considered a maximum resolution of 250 m, which is already very fine given the size of the GBR. We could nonetheless have considered even finer resolutions of e.g. 100 m or 50 m maximum resolution. The computational cost of running simulations with such resolution was however prohibitive. This means that we are not able to prove that the ocean circulation simulated on a 250 m resolution model are comparable with what would be obtained with even finer resolutions. As such, our best estimates of connectivity metrics, based on hydrodynamics modeled at 250 m, might be further improved. There are however limitations to the increase of the model resolution. On the one hand, models depend on several input data (bathymetry, coastlines, wind, and large-scale circulation forcings) which have a resolution that is often coarser than the model resolution. The gain in the model accuracy is hence offset by the accuracy of the forcings. On the other hand, the hydrodynamic model is based on physical assumptions that are only valid within a certain range of scales. If we increase the resolution beyond the range of scales prescribed by those assumptions, we would have to also modify the model’s mathematical formulation, and, for instance, consider a 3D model or even a fully non-hydrostatic model.

## Methods

### Study area

The GBR stretches over more than 2000 km along the north-eastern coast of Australia and is larger than 200 km on its widest part (Fig. 7). This coral reef ecosystem is located on the shallow continental shelf of Queensland, with a maximum depth of 200 m. In its official map of the GBR, the Great Barrier Reef Marine Park Authority (GBRMPA) identifies 3862 individual reefs, of which 3777 fall inside our modeling domain (the few reefs not taken into account in this work are either located north of the GBR in the Torres Strait, or beyond the shelf break). Reefs vary in shape and size, ranging from 0.01 to 100 km$^2$. Some are very close to each other, forming ribbons of reefs separated by narrow channels whose width can go down to $\sim$100 m. With its complex environment alternating between shallow reefs and deeper areas, modeling the hydrodynamics is particularly challenging.

### Ocean circulation model

**Figure 8:**
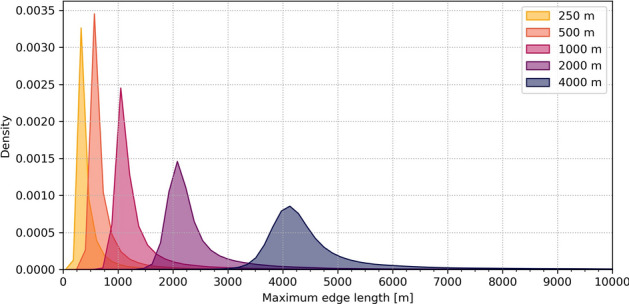
Kernel density plot of the maximum edge lengths of the elements composing the five meshes.

We use the 2D barotropic version of the multiscale coastal ocean model SLIM to simulate the currents and sea surface elevation on the whole GBR. To assess the effect of spatial resolution, we ran the model on five different unstructured meshes, with maximum resolutions of 250 m, 500 m, 1 km, 2 km and 4 km (Fig. 8). The meshes’ resolution varies in space and is the finest close to coral reefs, near coastlines, and in shallow areas. It progressively decreases elsewhere, to reach a minimum resolution of 25 km in the open ocean beyond the shelf break, far the coast and the reefs. The finest mesh contains more than $3~\times ~10^6$ triangular elements, whereas the coarsest one only consists of approximately $4~\times ~10^4$ elements. More details on the mesh generation and the model parametrization, as well as an extensive comparison of the meshes and the resulting differences in simulated hydrodynamics, can be found in Saint-Amand *et al.*^52^.

Increased spatial resolution allows for a better representation of the currents in topographically challenging areas. The model can hence explicitly represent small-scale features like eddies in the wake of reefs or increased velocities in the channels between reefs (Fig. 7c,d). With finer meshes, variations of the bottom drag between the reefs are also better reproduced, which leads to more variability in the currents as they are slowed down over reefs and accelerated in between. Depth variations between the reefs are better reproduced, which leads to more variability in the currents as they are slowed down over the reefs and accelerated in between. There are also depth variation over the reefs (Fig. 7b). Their influence on the currents can be reproduced with a sufficiently fine mesh (Fig. 7d) but not with a coarser one (Fig. 7c).

### Larval dispersal and connectivity

Larval dispersal simulations were initiated by releasing millions of ”virtual larvae” over all the reefs in the domain. Those larvae are then transported by the currents simulated on the five meshes. We model the physical processes driving larval dispersal thanks to a depth-averaged Lagrangian particle tracker as described in Hunter *et al.*^59^ and Spagnol *et al.*^60^. The advection term is modeled with a 4th-order Runge-Kutta scheme. The time step used for simulating larval dispersal is $\Delta t = 600$ s. The horizontal diffusivity coefficient *K* is calculated using Okubo^61^’s formulation.

We considered the mass spawning event of 2020 that started on 4 Dec. 2020 and simulated larval dispersal until 18 Feb. 2021, hence covering 75 days of dispersal. By the end of the simulation, nearly all the virtual larvae were either settled, dead or had left the domain. We considered two coral species with different larval life history traits: *Acropora millepora* and *Goniastrea retiformis*. *A. millepora* is a very common reef-building species that is present throughout the GBR. Figueiredo *et al.*^62^ have experimentally measured its mortality and competency acquisition rates, and its precompetency period. The mortality rate is set to 0.0540 day$^{-1}$ (corresponding to a life expectancy of 18.5 days) and the competency acquisition rate is set to 0.348 day$^{-1}$ after a precompetency period of 3.526 days. In other words, none of the larvae acquire the capacity to settle during the first 3.526 days of their life cycle, then some of them progressively acquire this ability at a rate of 0.348 day$^{-1}$. *G. retiformis* has been chosen for its contrasting biological properties. Connolly and Baird^63^ indeed experimentally estimated that *G. retiformis* larvae were instantaneously competent once released in the water column. This results in a vanishing precompetency period, while mortality rate was evaluated to 0.0870 day$^{-1}$ (corresponding to a life expectancy of 11.5 days), and the competency acquisition rate to 0.580 day$^{-1}$. The dispersal cycle of *G. retiformis* is hence shorter than for *A. millepora*, with quicker settlement and mortality. No loss of competency is assumed for both species, meaning that once larvae acquire the ability to settle, they never lose it. Although we limit our analysis to two species, a more comprehensive method would involve considering a complete range of possible values for each parameter. For clarity of results, however, we chose to restrict our analyses to two species that are fairly representative of the range of parameter values that would be possible for broadcast-spawning corals, particularly for pelagic larval duration.

During the three first nights of the simulation, millions of larvae are uniformly released over all the reefs between 6:00 p.m. and 12:00 a.m. AEST. The number of larvae released is proportional to the surface area of the reef with a constant density of 500 larvae/km$^2$ and a minimum release of 100 larvae for the smallest reefs. In total, about 12 million individual particles trajectories are simulated. This number of particles is sufficient to have stable connectivity estimates as we obtained the same results when using twice as many particles. Once released, larvae are transported by the currents. We assume that, after becoming competent, they will settle on the first reef they encounter, unless they die or leave the domain before that.

### Connectivity metrics

After 75 days of larval dispersal simulation, we obtain a connectivity matrix summarizing the number of larvae exchanged between any two reefs. From this matrix, the strength of the connection from reef *i* to *j* corresponds to the number of virtual larvae released on reef *i* and that settled onto reef *j*. This strength is denoted $C_{ij}$ where *i* is the row of the matrix corresponding to the source reef and *j* is the column, corresponding to the sink reef. When the entry $C_{ij}$ is non-zero, it means that there is a connection from reef *i* to reef *j*. Transposing this connectivity matrix into exploitable management information can be challenging. A reef map with 3000+ reefs leads indeed to a matrix with more than 9 million entries. This challenge is addressed by interpreting the connectivity matrix as a large graph where each reef is a node and each connection between any two reefs is a directed edge of the graph, with the number of larvae travelling from the start to the end of an edge corresponding to its strength (or *weight*). The connectivity matrix can then be analyzed with different graph-theory indicators and community detection algorithms.

We selected six connectivity indicators for their ability to highlight the effect of the model resolution on larval dispersal (Table 1). Those indicators either focus on what happens at the scale of the reef (identifying the most isolated and the most self-persistent reefs), or on their dispersion potential (identifying the best importers and exporters, both in terms of number and strength of connections). All those connectivity indicators are computed for all reefs and for the five different model resolutions. For each indicator, we also rank the reefs from low to high value, and identify the top 5% reefs with the highest values. These will be called ”hotspots”.

**Table 1:** Connectivity indicators selected to highlight differences in larval dispersal patterns between biophysical models with different resolutions.

Indicator	Description	Formula
Out-degree	Number of outgoing connections. It indicates to how many reefs a reef is sending larvae	$\displaystyle N_i^{out} = \sum \nolimits _{j:C_{ij} \ne 0} 1$
In-degree	Number of incoming connections. It indicates the number of reefs from which a reef is receiving larvae	$\displaystyle N_i^{in} = \sum \nolimits _{j:C_{ji} \ne 0} 1$
Mean outgoing strength	Mean strength of the connections starting from each reef, normalized by the total number of released larvae *n*. It indicates the average strength of outgoing connections	$\displaystyle K_i^{out} = \frac{\sum \nolimits _{j} C_{ij}}{N_i^{out} \times n}$
Mean incoming strength	Mean strength of the connections arriving at each reef, normalized by the total number of released larvae *n*. It indicates the average strength of incoming connections	$\displaystyle K_i^{in} = \frac{\sum \nolimits _{j} C_{ji}}{N_i^{in} \times n}$
Local retention	Proportion of larvae released over a reef which settle on the same reef^64^. It highlights the self-replenishment potential, or how selfish a reef is, by keeping its own larvae for itself. $S_i$ is the number of larvae released on reef *i*	$\displaystyle \lambda _i = \frac{C_{ii}}{S_i}$
Self-recruitment	Proportion of larvae settling on a reef which were released on the same reef^64^. It indicates reef isolation in the network by representing by how much a reef is its own source of larvae	$\displaystyle \sigma _i = \frac{C_{ii}}{\sum \nolimits _{j} C_{ji}}$

In addition to reef-scale indicators, we also identify clusters of reefs within the network. This was done by applying the *Strongly Connected Components* (SCC) community detection algorithm. This algorithm detects clusters of reefs exchanging many larvae together but being weakly connected with reefs not belonging to the same cluster. Such community detection methods are traditionally used to identify ecologically separated groups of reefs and hence infer the presence of barriers to larval dispersion between these groups^20^.

The SCC method^65,66^ only focuses on the presence of connections between reefs; it does not take the weight of those connections into account. This method groups reefs in the same community if they are *strongly connected*, i.e., if each reef of the community is reachable by any other reef of its community. The connection can potentially be satisfied through multiple steps in the graph. In other words, the SCC method identifies clusters of reefs between which loops of connections exist, ensuring there is a connection path between any two reefs of the same community. With this algorithm, a community can be formed as soon as two reefs exchange larvae in both directions. Conversely, there is also no maximum limit on the size of the communities: if a connection path exists between any two reefs, then the SCC algorithm output will be formed of only one community containing every reef of the network. The SCC algorithm aims to detect groups of reefs presenting lots of bidirectional connections, and being as such genetically well mixed.

## Supplementary Information

Supplementary Figures.

## Data Availability

The reef map used in this work as well as the connectivity matrices are available on the Open Science Framework repository: 10.17605/OSF.IO/C39VK.

## Code availability

The SLIM model source code can be found at https://git.immc.ucl.ac.be/slim/slim.
