# Supplementary material for: Biophysical models resolution affects coral connectivity estimates
Source: Sci Rep. 2023 Jun 9;13:9414. doi: 10.1038/s41598-023-36158-5 (PMC10256739; doi:10.1038/s41598-023-36158-5)
Supplement: Supplementary file 1 — Supplementary Figures. [file 41598_2023_36158_MOESM1_ESM.pdf]

Supplementary material: Spatial distribution of the connectivity indicator hotspots along the GBR

Antoine Saint-Amand, Jonathan Lambrechts, Emmanuel Hanert

Figures S.1 and S.2 present, for *A. millepora* and *G. retiformis* respectively, the spatial distribution along the GBR of the reefs presenting the 5% highest values of the six connectivity indicators covered by this study. For the sake of readability, only the hotspots detected at 250 m and 4 km resolution are presented. In- and out-degree and mean strength hotspots are mostly located in the same part of the GBR, respectively in the southern part (in/out-degree), and in the central and northern part (mean strength). The distribution of local retention and self-recruitment hotspots is more scattered along the GBR. Those observations hold for both studied species.

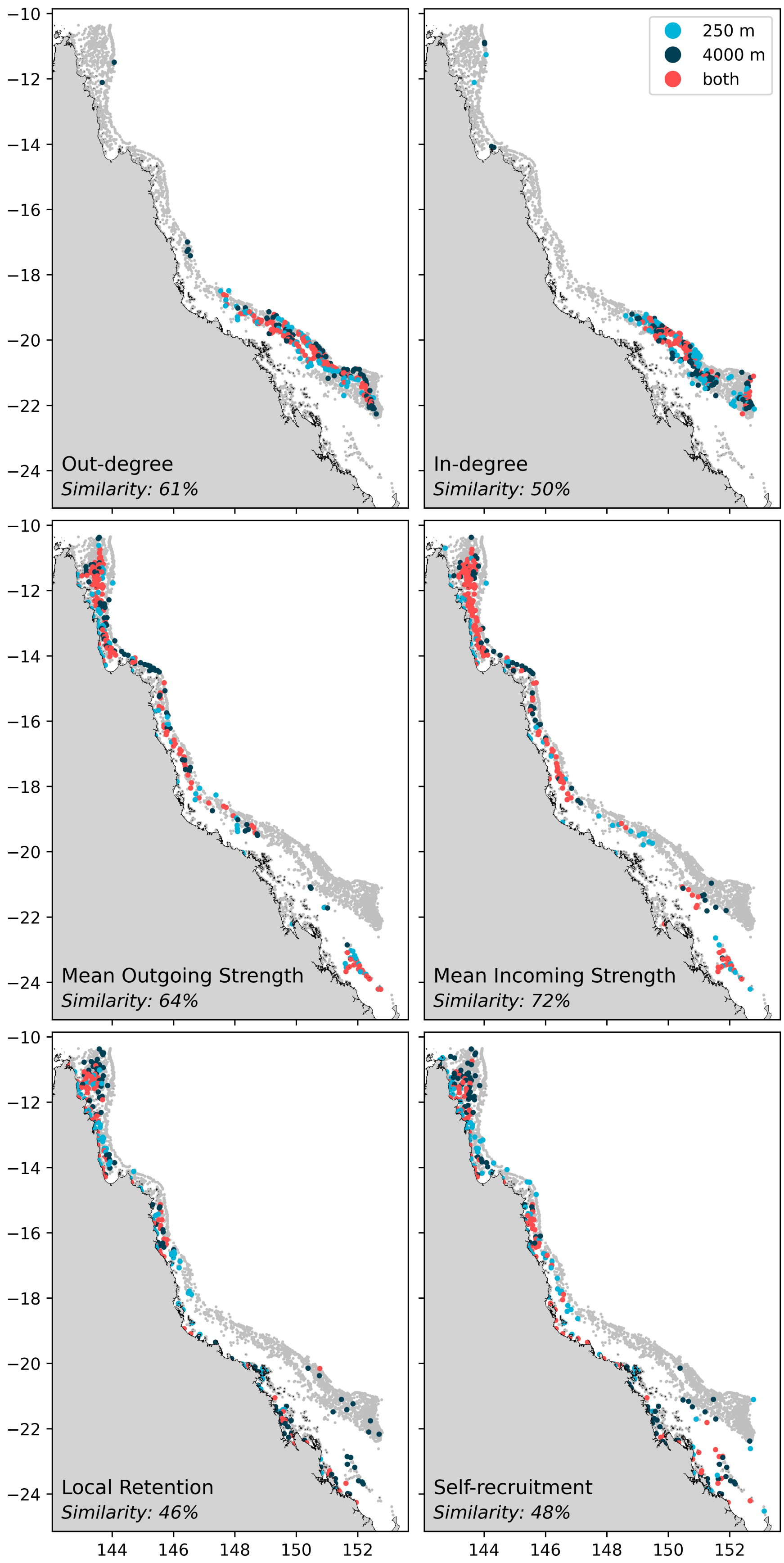

Figure S.1: Spatial distribution of the top 5% connectivity indicator hotspots for *A. millepora*. The colour coding indicates if the reefs are among the hotspots for the 250 m and/or on the 4 km model resolutions. The displayed similarity gives the percentage of hotspots common for both model resolutions.

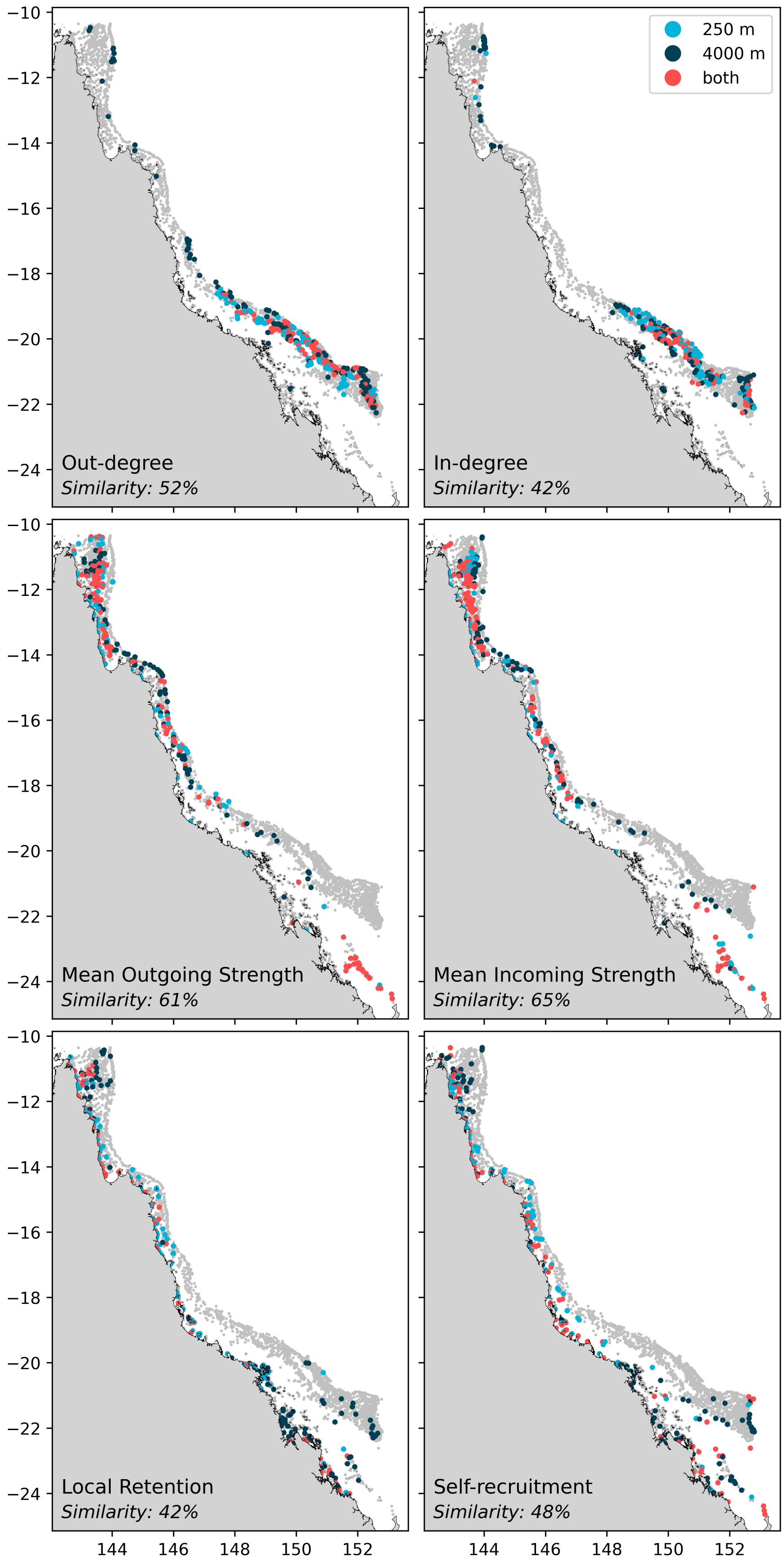

Figure S.2: Spatial distribution of the top 5% connectivity indicator hotspots for *G. retiformis*. The colour coding indicates if the reefs are among the hotspots for the 250 m and/or on the 4 km model resolutions. The displayed similarity gives the percentage of hotspots common for both model resolutions.
